# Tardive dystonia improved with discontinuation of trazodone in an elderly schizophrenia patient: a case report

**DOI:** 10.1186/s12991-020-00273-8

**Published:** 2020-04-01

**Authors:** Yoshinori Kadota, Hikaru Hori, Michiko Takayama, Chikako Okabe, Naotoshi Ohara

**Affiliations:** 1 Minamigaoka Hospital, 3-13-1 Imamachi, Kokurakita-ku, Kitakyushu, Fukuoka 8030862 Japan; 2 Department of Psychiatry, University of Occupational and Environmental Health, 1-1 Iseigaoka, Yahatanishi-ku, Kitakyushu, Fukuoka 8078555 Japan

**Keywords:** Trazodone, Tardive dystonia, Schizophrenia, Delirium, Insomnia

## Abstract

**Background:**

Tardive dystonia associated with antidepressant use is rare and often under-recognized. We had an experience with trazodone, which is used for delirium and insomnia prescribed in general hospital, inducing tardive dystonia.

**Case presentation:**

A 61-year-old Japanese woman had been treated for schizophrenia. She was moved to general hospital because of consciousness disturbance. She was prescribed trazodone (25 mg/day) for delirium and insomnia. After she was discharged, she returned to the psychiatric hospital with tardive dystonia. Her dystonia symptoms improved with 3 days of discontinuing trazodone.

**Conclusion:**

In the present case, long-term use of trazodone induced tardive dystonia. Discontinuing trazodone rapidly improved tardive dystonia.

## Background

Antipsychotics, which are dopamine antagonists and sometimes antidepressants, cause tardive dystonia [1]. However, tardive dystonia associated with antidepressant use is rare and often under-recognized [2].

Despite the lack of relevant and reliable safety data, trazodone continues to be widely used for treating delirium and insomnia [3–6]. Some clinicians have been noted to unnecessarily prescribe trazodone to patients with tardive dystonia.

Here, we describe the case of a Japanese woman with schizophrenia who was treated with risperidone and developed tardive dystonia after the addition of trazodone to her regimen.

## Case presentation

A 61-year-old, right-handed, Japanese woman had been treated for schizophrenia since her mid-20s. She had no family history of head trauma, seizures, or substance abuse and received haloperidol, risperidone, and olanzapine for the treatment of her psychotic symptoms, such as excitement, hallucination, and difficulty in controlling emotions. Her schizophrenia symptoms indicated partial response to treatment; however, she complained of decreased motivation, delusions, and convulsive symptoms and was therefore admitted to a psychiatric ward 5 years ago. One year ago, she was moved from our psychiatric ward to a general hospital because of water intoxication-induced consciousness disturbance. At this point she was treated with risperidone 6 mg/day and chlorpromazine 75 mg/day.

The patient was discharged from the general hospital after the treatment of water intoxication and consciousness disturbance. She was prescribed trazodone (25 mg/day) for delirium and insomnia at the time of discharge. According to her chart, she had not received calcium channel blockers, such as flunarizine or cinnarizine, or lithium over the past 2 years. Within a week of discharge, she returned to the psychiatric ward with complaints of general slowness and excessive fatigue. She exhibited dysphagia, and the nurses had to regularly monitor her during her meals. The abovementioned symptoms gradually progressed and caused considerable distress in her communication, impairing her social and occupational functions.

**Fig. 1:**
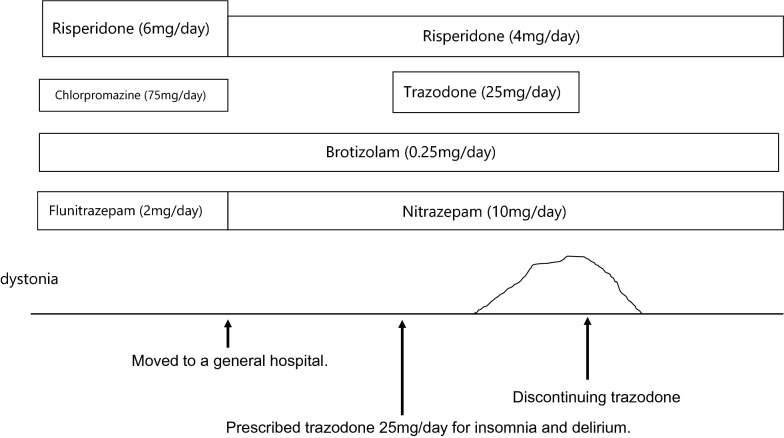
Clinical course of trazodone-associated tardive dystonia

Her hematological and biochemical examinations as well as thyroid function (free total thyroxine and thyroid-stimulating hormone) were normal. Regarding the psychiatric history and diagnostic check, neither conversion nor malingering disorder was relevant for the patient. Secondary tardive dystonia caused by trazodone was diagnosed. Her dystonia symptoms, including cervical extension and dysphagia, improved within 3 days of discontinuing trazodone (Fig. 1).

## Discussion

Here, we report a case of tardive dystonia caused by administering trazodone along with the antipsychotic risperidone (4 mg/day). Few studies on trazodone-induced extrapyramidal symptoms, such as dystonia, have been reported to date [7–10].

Trazodone is widely used for treating delirium and insomnia, particularly in the elderly. Trazodone is a potent antagonist of the 5-HT2A and 5-HT2c receptors, a weak inhibitor of serotonin reuptake, a moderately to highly potent α-adrenoceptor antagonist (particularly to α1), and a moderate histaminergic (H1) antagonist.

The pathophysiology of dystonia remains unclear owing to lack of data supporting any particular mechanism. Because tardive dystonia is most commonly caused by antipsychotics, postsynaptic supersensitivity induced by sustained inhibition of dopaminergic neurotransmission was speculated to be the major mechanism underlying its clinical presentation. However, the contrast between the benefits of anticholinergics for tardive dystonia and the deterioration of patients with tardive dyskinesia following treatment with such drugs indicates a difference in their pathophysiology. Other mechanisms have also been suggested; increased serotonin transmission may inhibit dopaminergic neurons in the ventral tegmental. In addition, Remington reported a possible role of serotonergic and noradrenergic modulation of the cholinergic pathway in tardive dystonia and Pisa syndrome [11]. Another theory involves calcium channel blockers, which reportedly cause tardive blepharospasm [12] and Parkinsonism [13]; however, a review reported that the effects of calcium channel blockers on antipsychotic-induced tardive dystonia are unknown [14]. Trazodone was recently demonstrated to inhibit T-type calcium channels [15]; however, the mechanism underlying how this effect contributes to tardive dystonia remains to be elucidated.

Clinicians have been sometimes known to unnecessarily prescribe trazodone for insomnia and delirium. In the present case, long-term use of trazodone induced tardive dystonia. Discontinuing trazodone rapidly improved tardive dystonia.

## Conclusion

Using trazodone as a hypnotic can cause tardive dystonia in elderly patients with schizophrenia. All healthcare providers should be informed of this serious but seemingly completely reversible adverse effect.

## Data Availability

All data generated or analyzed during this study are included in this published article.
